# Optical Band Gap Alteration of Graphene Oxide *via* Ozone Treatment

**DOI:** 10.1038/s41598-017-06107-0

**Published:** 2017-07-25

**Authors:** Md Tanvir Hasan, Brian J. Senger, Conor Ryan, Marais Culp, Roberto Gonzalez-Rodriguez, Jeffery L. Coffer, Anton V. Naumov

**Affiliations:** 1 Department of Physics and Astronomy, Texas Christian University, Fort Worth Texas, USA; 2 Department of Chemistry and Biochemistry, Texas Christian University, Fort Worth Texas, USA; 3 Department of Physics, Saint Mary’s University of Minnesota, Winona Minnesota, USA

## Abstract

Graphene oxide (GO) is a graphene derivative that emits fluorescence, which makes GO an attractive material for optoelectronics and biotechnology. In this work, we utilize ozone treatment to controllably tune the band gap of GO, which can significantly enhance its applications. Ozone treatment in aqueous GO suspensions yields the addition/rearrangement of oxygen-containing functional groups suggested by the increase in vibrational transitions of C-O and C=O moieties. Concomitantly it leads to an initial increase in GO fluorescence intensity and significant (100 nm) blue shifts in emission maxima. Based on the model of GO fluorescence originating from sp^2^ graphitic islands confined by oxygenated addends, we propose that ozone-induced functionalization decreases the size of graphitic islands affecting the GO band gap and emission energies. TEM analyses of GO flakes confirm the size decrease of ordered sp^2^ domains with ozone treatment, whereas semi-empirical PM3 calculations on model addend-confined graphitic clusters predict the inverse dependence of the band gap energies on sp^2^ cluster size. This mod﻿el explains ozone-induced increase in emission energies yielding fluorescence blue shifts and helps develop an understanding of the origins of GO fluorescence emission. Furthermore, ozone treatment provides a versatile approach to controllably alter GO band gap for optoelectronics and bio-sensing applications.

## Introduction

Due to its growing role in industry a functional derivative of graphene, graphene oxide (GO) has become a subject of active scientific inquiry. GO is a prospective material for multiple optoelectronics applications such as high luminance organic light-emitting diodes^1^, organic solar cells^2^, chemical sensors^3^, and flexible transparent electronics^4^. Although it is a transformed form of graphene, graphene oxide still possesses high transparency^5^, significant conductivity^6^, and high mechanical strength^7–9^, comparable to those of graphene. In addition to these properties, GO is water-soluble and exhibits fluorescence, which makes it a more attractive candidate than graphene for optoelectronics and biomedical applications^10, 11^. Different GO fabrication methods including Hummers/modified Hummers methods^12, 13^, facile oxidative techniques utilizing benzyl peroxide^14^, and structure-defining synthetic methods^15–17^ result in GO materials with different optical and electronic properties that are strictly defined by the synthetic procedure. This restricts potential applications of GO to those fitting with the properties of as-synthesized materials. At the same time, a variety of oxygen addend structures^18^ and their arrangements o﻿n GO surface﻿ give promise for modification of GO optical properties.

The ability to tune the GO band gap is highly desired in energy storage/conversion devices. For example, varying the electronic gap of GO-based active material in solar cells requiring high power conversion efficiencies will allow controlling the performance of the device, mainly over the open circuit voltage and short circuit current^19^. The band gap tunability of GO may also allow its use in mid-IR range photodetectors^20^ and ultrafast lasers in a form of a saturable absorber^10^ superseding the performance of graphene. A number of techniques such as chemical modification^10, 21, 22^, infrared irradiation^23^, thermal exfoliation^24^, exfoliation of GO using focused solar radiation^25^, photoreduction^26^, photothermal deoxygenation^27^, flash reduction^28^, laser-induced reduction^29, 30^, photocatalytic reduction^31^ and mechanical compressive strain processing^32^ are known to alter GO band structure. However, many of those methods are difficult to control due to their complexity, and do not allow the fine tuning of the gap energy for specific applications. Here, we aim to develop a reliable method to surmount those difficulties, and at the same time achieve controlled alteration of the GO optoelectronic properties. Previously ozone treatment has been utilized to modify the fluorescence intensity of graphene oxide^33, 34^; however, substantial band gap alteration of GO has not been reported to date. In this work, for the first time, we show that GO band gap can be significantly modified by timed ozone treatment. This solution-based process, as opposed to previously developed structural modification methods, is exceptional in its simplicity and high degree of control. As a result, it provides a route to tailor the electronic properties of GO for such applications as polymer tandem solar cells^35^, energy storage devices^36^ and solid-state electric double layer transistors^37^. PM3 semi-empirical simulation of stepwise GO oxidation attributes band-gap alteration to ozone-induced structural modification of sp^2^ graphitic islands in GO. This model, supported by FTIR and TEM analysis of GO flake composition, provides an insight into the structural origins of GO fluorescence and allows for optical characterization of GO structure.

## Results and Discussion

**Figure 1:**
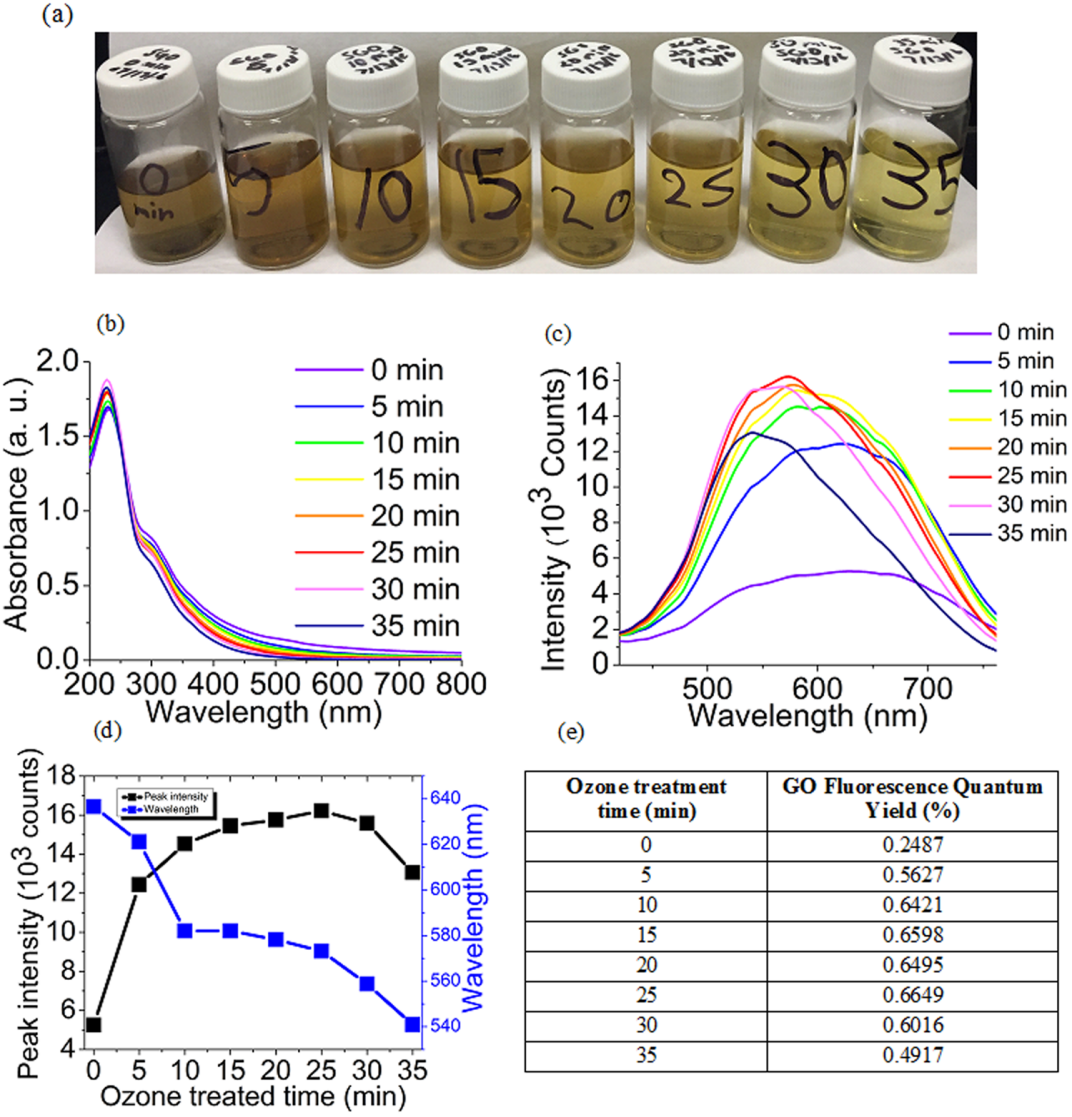
(**a**) Pictures of the ozone-oxidized GO samples with respective oxidization times (0, 5, 10, 15, 20, 25, 30, 35 minutes) (**b**) Absorption spectra of 0 to 35 min ozone-treated GO (**c**) Fluorescence spectra of 0 to 35 min ozone-treated GO (**d**) A plot of fluorescence intensity and maximum emission wavelength variation with ozone treatment (**e**) The table of GO fluorescence quantum yields at particular ozone treatment times.

Based on previous theoretical works we expect the band gap in GO to be dependent on its physical structure, i.e. the types of oxygen-containing functional groups or their arrangements^38–40^. In order to alter the optical band gap, we subject aqueous suspensions of graphene oxide to oxidizing ozone treatment inside the ultrasonic bath for the periods of 0 to 35 minutes which was previously utilized to convert RGO (Reduced Graphene Oxide) into GO^17^. Ultrasonic treatment serves as a means of disaggregation, improving the access of ozone to GO sheets. Even though we observe that after 10–30 minutes of ozone treatment, GO samples become somewhat more transparent (Fig. 1(a)), this mild color change does not result in significant variations in the UV – visible absorption spectra (Fig. 1(b)). Absorption features typical to single-layer GO suspensions [21, 22], including the 228 nm peak, corresponding to π→π^*^ transition of sp² carbon^41–44^, remain the same for all the ozone-treated samples, with a negligible shift in peak position (1–2 nm) and only slight variations in intensity. From the absence of significant changes in absorption spectra, it follows that ozone treatment does not strongly alter the chemical structure of the dominant absorbing species on GO sheets.

**Figure 2:**
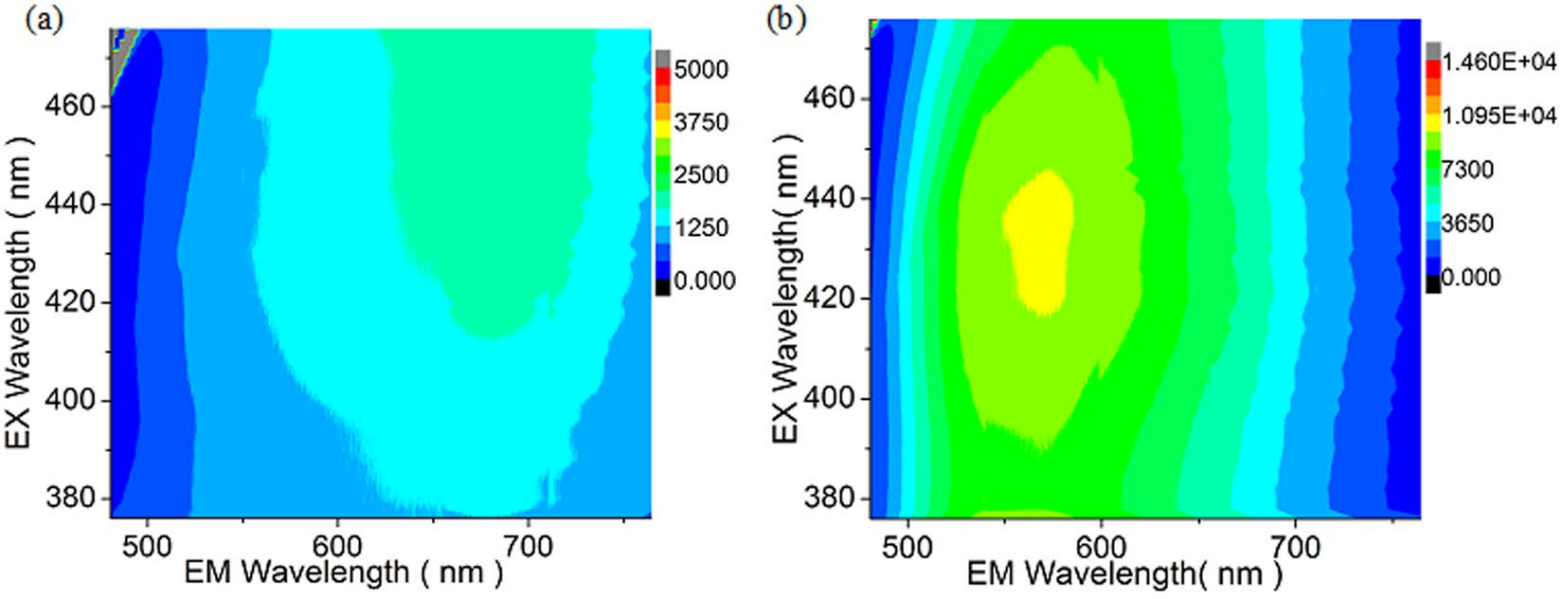
Photoluminescence excitation (PLE) contour graphs of (**a**) untreated and (**b**) 30 min ozone treated GO samples. X and Y axis represent emission and excitation wavelengths, respectively.

Unlike absorption, GO fluorescence excited at 400 nm shows substantial spectral changes with ozone treatment (Fig. 1(c)). With timed ozone exposure, the intensity of the broad red/near-IR GO fluorescence feature increases gradually (from 0 to 25 min of treatment) and exhibits a large blue shift up to a 100 nm from deep red into green. This shift suggests an increase in GO optical band gap with functionalization, or the conversion of the lower-energy fluorescing species into higher energy ones. The decreased spectral width, on the other hand, may point toward narrowing the size distribution of the fluorescing species, as wide GO emission peaks are often attributed to a broad range of emissive cluster sizes^38, 41, 45^. Photoluminescence excitation (PLE) experiments show a similar trend: an increased emission intensity, blue shifts in emission maxima with ozone treatment and also blue shifts in excitation maxima (Fig. 2). Although absorbance spectra experience little change with ozone treatment indicating no drastic variations in general GO structure, the shifts in excitation peak position point to structural alteration of emissive species specifically. Based on these pronounced spectral changes, we suggest that ozone treatment may alter the amount, type, and/or the arrangement of functional groups in GO, affecting its optoelectronic properties.

The degree of structural changes varies with ozone treatment: a longer 35 min processing results in a significant decrease in emission intensity due to over-oxidization. Prolonged ozone treatment is known to induce GO flake scission^17^ and, potentially, a decomposition of fluorescing centers causing a decrease in GO emission. Thus, we expect two competing processes to take place: (1) the addition and rearrangement of functional groups on GO surface resulting in a blue shift, increase in the emission intensity and fluorescence quantum yield (Fig. 1(d),(e)), and (2) deterioration of GO flakes, leading to fluorescence quenching. Generally, the increase in the emission intensity accompanied by blue shifts is uncommon for chemical functionalization ﻿of GO, as the introduction of additional oxidative defects is expected to produce quenched red-shifted emission^46, 47^. This suggests that the observed phenomenon is likely based on the variations in electronic structure induced by changes in functional group arrangements rather than a random defect formation.

**Figure 3:**
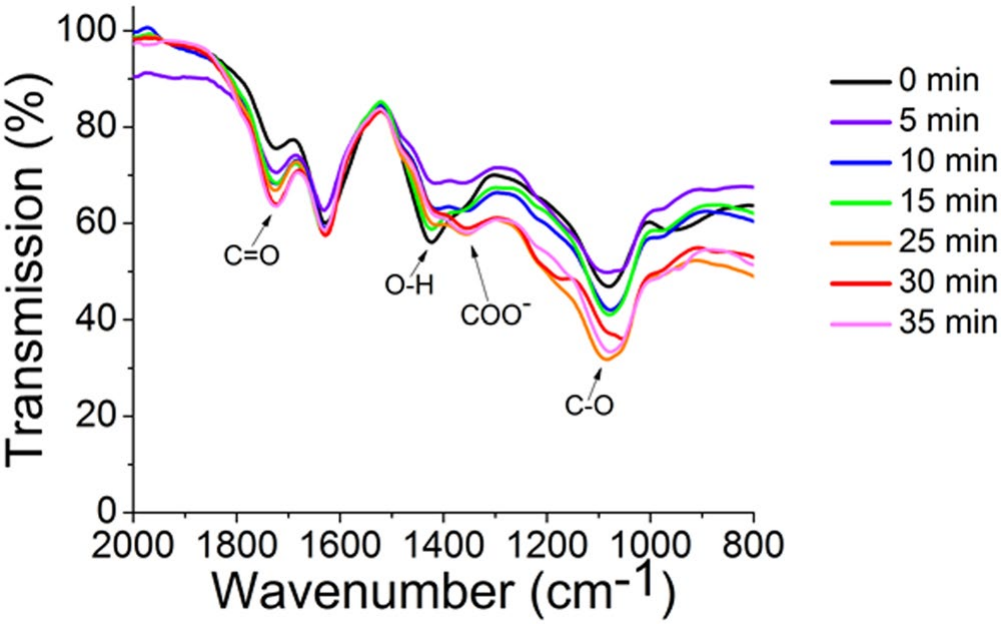
The IR spectra of ozone-treated GO. Observed transitions include C-O stretch (~1085 cm^−1^), COO^−^ stretch in COOH group (~1360 cm^−1^), C-OH bend/-O-H deformation (~1425 cm^−1^), and C=O stretch in COOH group (~1725 cm^−1^).

We utilize infrared spectroscopy of the ozone-treated samples to follow the structural modification of GO and assess its composition. Untreated GO (0-min sample) shows a number of vibrational transitions in its IR spectra (Fig. 3) corresponding to oxygen-containing functional groups that split graphitic sp^2^ network into individual islands. Those groups include C-O stretch (~1085cm^−1^), C-OH bend/-O-H deformation (~1425cm^−1^), and a weak C=O stretch in the COOH group (~1725cm^−1^). With timed ozone oxidation, O-H features become less prominent, whereas the transmittance intensity of transitions corresponding to C-O moieties, C=O stretch of carboxylic groups and COO- peak increase. Unlike O-H groups that may be present at any sp^3^-hybridized carbon, carboxylic addends can be only formed either at the edges or require substantial defect formation for functionalization of internal lattice carbon atoms. Thus, the observed enhancement of the vibrational transitions suggests potential changes in both functional group types and GO surface morphology: ozone treatment has likely introduced additional splitting of sp² network due to the fomration of some new and/or rearrangement of already existing functional groups.

We also anticipate that some oxidative debris (OD) may be present in our GO sample as per^48^ or generated due to ozone treatment. The absorption spectra of OD are markedly different from that of GO as it has been previously shown in^16^. Therefore, in the event of the increase in the OD content due to ozone treatment we would anticipate the corresponding change in GO absorption spectra. As no substantial absorption changes were detected (Fig. 1b), we consider that there was no significant change to the amount of OD in the sample during the ozone treatment. OD contribution to fluorescence spectra has been shown in^16^ to be significant mostly at concentrations requiring substantial alteration of the absorption spectra of GO; thus, ozone-induced changes in the emission observed here are not attributed to OD. These oxidation-induced optical changes can be potentially explained within the framework of several existing theoretical models of GO fluorescence. Between the two most prominent scenarios, the first one considers emission originating from localized electronic states surrounding individual functional groups. Due to electronic confinement, those may produce a band gap in GO enabling fluorescence emission^39–41, 49^. In principle, this model can explain the increase in emission intensity upon ozone treatment due to the introduction of new functional groups’-confined fluorescence centers. However, it does not justify the blue shift. The second theory attributes the emission in GO to the regions of graphitic carbon confined by the encircling functional groups. Quantum confinement in such areas is expected to produce a band gap in GO that depends inversely on the size of the regions^38, 46, 50^. Considering a potentially wide distribution of graphitic region sizes, we initially expect a broad emission from GO, which is the case in Fig. 1(c).

Within this theoretical framework the decreases in spectral width and oxidation-induced blue shifts (Fig. 1(c)) can be explained by the band gap increase while larger graphitic domains get broken down into smaller fragments with the introduction of new functional groups. Blue shifts of excitation spectral maxima evident from PLE maps (Fig. 2 and Figure S2) also point to the increase in the band gap/decrease in the size of the excited domains. Similar trend with blue shifted emission was previously reported as a result of the appearance of small graphitic domains originating in highly oxidized GO upon the reduction of the functional groups^43, 51^. These observations complement the proposed model considering that further reduction observed in^43, 51^ induced red shifts due to the agglomeration of the small graphitic regions into larger ones: a reverse process to oxidation-induced splitting of sp^2^ domains proposed in the present work.

**Figure 4:**
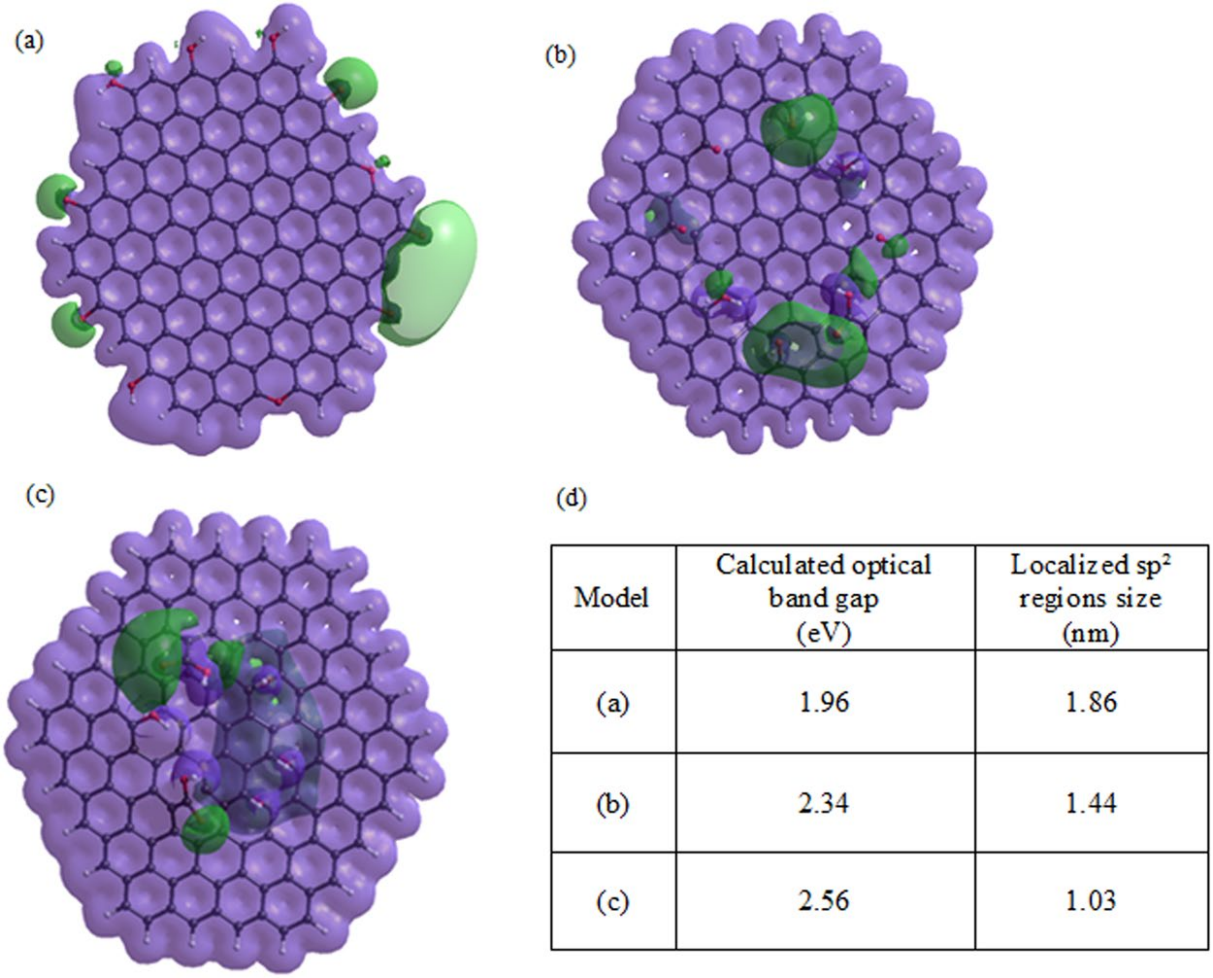
Computation of electrostatic potential of graphene sheet fragment with C=O, C-OH and C-O-C groups surrounding the region of graphitic carbon ﻿﻿﻿of﻿ the size of (**a**)﻿ 1.86 nm (**b**) 1.44 nm﻿ and (**c**) 1.03 nm. White, black and red atoms represent hydrogen, carbon, and oxygen, respectively for all the models. Green color represents negative electrostatic potential around the functional groups, whereas the purple color represents constant potential isosurfaces. (**d**) The table of calculated optical band gaps for the models (a), (b) and (c).

**Figure 5:**
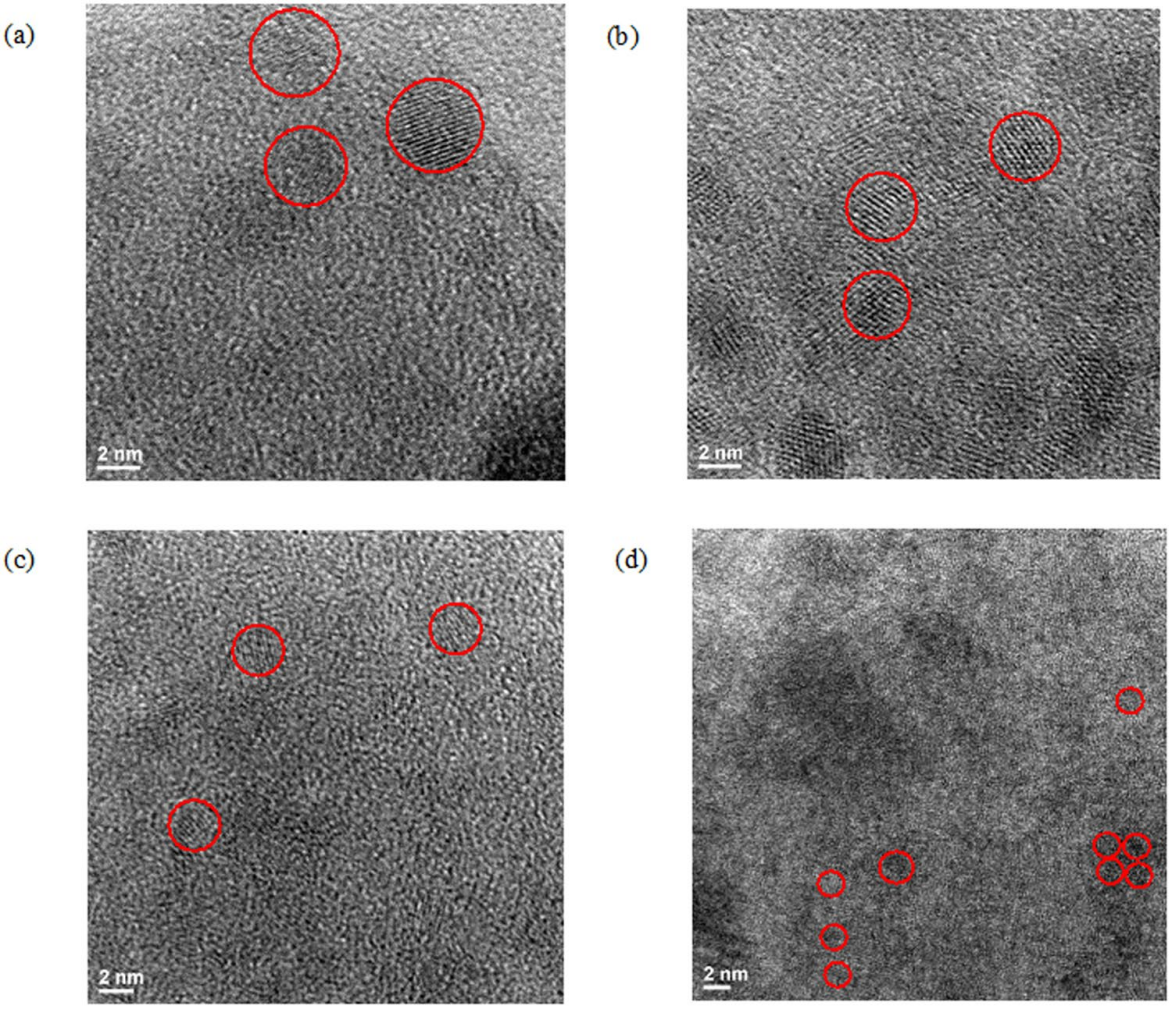
TEM images for (**a**) 0 min (**b**) 10 min (**c**) 15 min & (**d**) 30 min ozone treated samples. The estimated average graphitic carbon cluster sizes for these samples are 3.69, 1.85, 1.73, and 1.28 nm, respectively. Representative regions are circled in red, however all ordered sp2 regions were considered in the statistics.

In order to verify the predictions of this graphitic cluster-centered theory of GO emission, we model oxidation-induced changes in the size of sp^2^ graphitic islands and their effect on GO optical response. We introduce three HyperChem models of graphitic carbon sheets each containing a region of sp² carbon surrounded by functional groups and calculate their electronic configuration using a PM3 semi-empirical approach. In each consecutive model the size of the graphitic island was decreased by the introduction of additional COOH groups (as the increase in COOH group content was one of the most evident changes in FTIR spectra). All three models show the regions of negative electrostatic potential surrounding the functional groups and encircling the sp^2^ carbon islands (Fig. 4), which serves to support the confinement theory. The band gaps of the modeled fragments exhibit a monotonic increase with the diminishing size of the graphitic island. This observed trend (Fig. 4(d)) akin to theoretical results of Kozawa *et al*.^38^ for plain carbon nanodiscs directly follows blue shifts in GO emission introduced by ozone treatment. Such agreement supports the idea of ozone-induced size alteration of sp^2^ domains affecting fluorescence emission in GO. Although these models are simplistic and neglect the excitonic contribution^38^, in conjunction with experimental data, they provide grounds to explain emission in GO and its modulation due to ozone-induced structural alteration. In order to support the aforementioned theoretical predictions, we utilize TEM imaging of ordered graphitic substructures in GO flakes subjected to 0, 10, 15 and 30 minutes of ozone treatment (Fig. 5). The statistics of over 150 ordered graphitic clusters with well-defined lattice observed in TEM images indicate the monotonic decrease of average cluster size from 3.69 nm down to 1.28 nm with ozone treatment (Fig. 5). This apparent trend verifies the alteration of graphitic islands with ozone-induced functionalization and supports the proposed theoretical model of the sp^2^ region size-dependent optical band gap in GO.

Based on this rationale, we infer that initially broad fluorescence signature given by wide size distribution of graphitic islands in GO gets narrowed and blue-shifted upon ozone treatment due to the depletion of the larger graphitic regions into smaller fragments. These structural modifications lead to the tuning of the GO band gap, which has a promising potential ﻿for optoelectronics applications in polymer solar cells, and energy conversion devices^19^.

## Conclusion

In this work, for the first time, we report controllable ozone-induced modification of GO band gap in aqueous suspension. Although absorption features show little change with continuous ozone treatment, a gradual increase in emission intensity and substantial blue shifts in fluorescence spectra are observed. These optical changes suggest an alteration of the band gap in GO due to structural modifications introduced by the addition and/or rearrangement of functional groups. Within the framework of GO emission model attributing fluorescence to confined graphitic islands, we expect the addition of new oxygen addends to split the regions of graphitic carbon into smaller segments, thus increasing a confinement-defined band gap. Semi-empirical modeling of GO flakes with different graphitic island sizes describes this scenario providing band gap values that follow the trend of experimental emission energies. The decrease of graphitic cluster size with ozone treatment is further supported by TEM statistical analysis. Thus, a controllable variation of GO optoelectronic properties observed in this work helps elucidate the mechanism of GO emission and also warrants a capability of adjusting GO optical response to fit multiple optoelectronics applications. Modifiable electronic properties are critical for the use of GO as a versatile material in liquid crystal displays^52^, broadband optical limiters^10^ and biosensors^53^ etc. facilitating the advancement of photonics and optoelectronics.

## Methods

### Sample Preparation

Single-layered GO purchased from GooGraphene was used for ozone treatment. Prior to ozone-oxidation, 2 mg of single-layered GO was dispersed in 15 ml of water by direct probe ultrasonic treatment for 30 min at 3 W. This yielded dark yellow/light brown colored suspensions. Ozone produced by Enaly (Model: 5000BF-1 3 g/L) ozone generator fed by oxygen source was further introduced to these GO suspensions at 40% of maximum ozone level (~1.2 g/L) under low power agitation in the ultrasonic bath intended to split aggregated flakes. Ozone processing was carried out for the periods of 0 to 35 minutes in 5 minute time intervals; fluorescence and absorption were measured for each time point. To calculate the quantum yield of ozone treated and untreated GO we follow a comparative method^54^ where coumarin-153 (47% quantum yield in ethanol at 400 nm excitation^55^) was chosen as a convenient reference material with similar excitation and emission wavelengths.

Samples for Transmission Electron Microscopy (TEM) analysis were prepared from a drop of ozone-treated GO in aqueous suspensions, dried on a carbon-coated 200-mesh copper grid under ambient conditions. Untreated GO was deposited on carbon conducting tape to perform the SEM characterization.

### Optical measurements and characterization of GO samples

GO fluorescence spectra and PLE maps were recorded using Horiba Scientific, SPEX NanoLog fluorescence spectrofluorometer. In order to measure fluorescence spectra, 400 nm excitation was chosen on the basis of previous works^17^. In PLE experiments, excitation wavelength was scanned from 376 to 476 nm with 2 nm interval. Agilent Technologies, Cary 60 UV-Vis spectrometer was utilized to measure the absorbance of GO in suspensions diluted by the factor of 5 in the range of 200–800 nm. In order to determine the specific oxygen functionalities and their relative abundance in GO, ozone-treated samples were freeze-dried in Labconco, FreeZone 4.5 freeze-dryer and analyzed via the ATR mode of Thermo Nicolet Nexus, 670, FTIR. The size of the sp² graphitic carbon clusters was studied *via* TEM using a JEOL-JEM2100 instrument operating at 200 kV. We analyzed over 25 images to provide reliable statistics of graphitic carbon cluster sizes (diameters) within the flakes of GO ozone-treated for 0, 10, 15 and 30 min. The cluster size was identified by outlining (inscribing) the boundaries of each region in the ImageJ program that has further computed the area of the outlined segment. We studied the topology of the untreated GO utilizing SEM (JEOL, JSM-7100F) analysis which qualitatively show substantial amounts of monolayer GO flakes. Additionaly, the vendor (Goographene) has shown in their AFM analysis that the starting GO materials contain 99% monolayer ratio with 0.7–1.2 nm in thickness of each layer^56^.

## Electronic supplementary material

Supporting Information
