# Supplementary material for: Optical Band Gap Alteration of Graphene Oxide via Ozone Treatment
Source: Sci Rep. 2017 Jul 25;7:6411. doi: 10.1038/s41598-017-06107-0 (PMC5527010; doi:10.1038/s41598-017-06107-0)
Supplement: Supplementary file 1 — Supporting Information [file 41598_2017_6107_MOESM1_ESM.pdf]

# Supplementary Information

## Optical Band Gap Alteration of Graphene Oxide via Ozone Treatment

*Md. Tanvir Hasan[1], Brian J. Senger[1], Conor Ryan[1], Marais Culp[3], Roberto Gonzalez-Rodriguez[2], Jeffery L. Coffer[2], *Anton V. Naumov[1]*

[1]Department of Physics and Astronomy, Texas Christian University

[2]Department of Chemistry and Biochemistry, Texas Christian University

[3]Department of Physics, Saint Mary's University of Minnesota

**E-mail: a.naumov@tcu.edu*

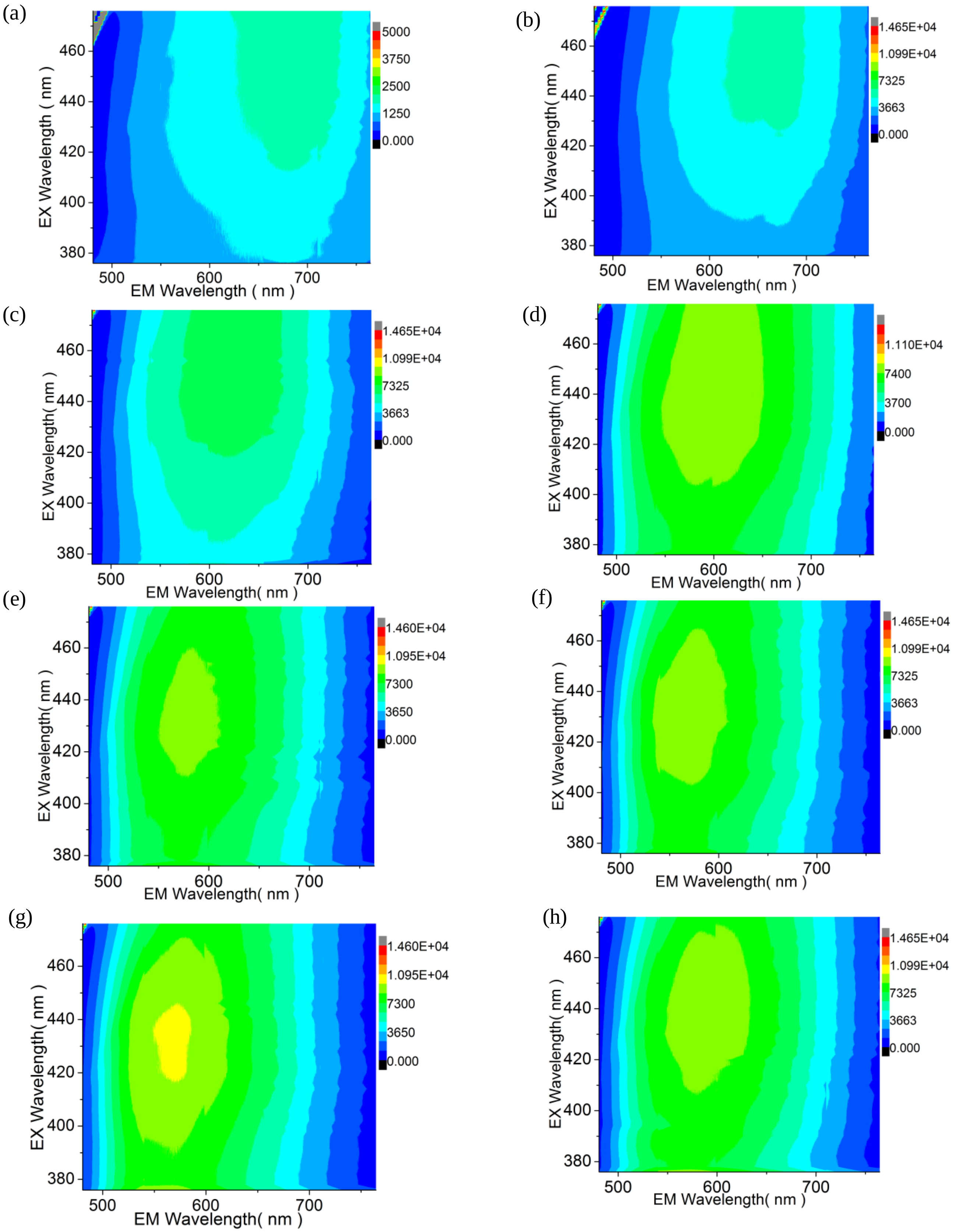


**Figure S1.** Photoluminescence excitation (PLE) maps of (a) 0 min (b) 5 min (c) 10 min (d) 15 min (e) 20 min (f) 25 min (g) 30 min (h) 35 min ozone treated GO samples. X and Y axis represent the emission and excitation wavelength,

Figure S1(a-h) represents the PLE contour graphs of 0, 5,10, 15, 20, 25, 30 and 35 minutes ozone treated samples, respectively. With ozone treatment, both excitation and emission maxima experience a gradual trend of blue shifts except for 35 min-treated sample, optical changes in which could be attributed to overoxidation-induced deterioration of GO sheets[1].

SEM of the starting GO material shows the abundance of single layer graphene flakes (Figure S2).

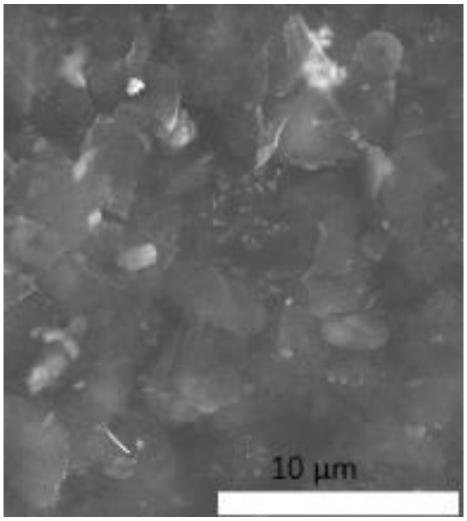


**Figure S2.** SEM image of single-layer flakes in the starting Graphene Oxide material

**Reference:**

1 Md Tanvir, H. *et al.* Modifying optical properties of reduced/graphene oxide with controlled ozone and thermal treatment in aqueous suspensions. *Nanotechnology* **28**, 065705, doi:10.1088/1361-6528/aa5232 (2017).
